# Peripheral primitive neuroectodermal tumor: a rare case of peripheral facial paralysis

**DOI:** 10.1016/S1808-8694(15)31139-3

**Published:** 2015-10-20

**Authors:** Romualdo Suzano Louzeiro Tiago, Marcio Ricardo Barros Pio, Marcelo Nery Silva, Lupércio Oliveira do Valle

**Affiliations:** 1 PhD in Sciences – Post graduation in Otorhinolaryngology – Head and Neck Surgery - Universidade Federal de São Paulo. Assistant physician – Otolaryngology Department - Hospital do Servidor Público Estadual de São Paulo e do Hospital do Servidor Público Municipal de São Paulo; 2 ENT Resident (2nd year) – Otorhinolaryngology Department -Hospital do Servidor Público Municipal de São Paulo; 3 Assistant Physician – Neurosurgery Department - Hospital do Servidor Público Municipal de São Paulo; 4 M.S. in Human Communication Disorders - PUC-SP, Assistant Physician at the Otorhinolaryngology Department - Hospital do Servidor Público Municipal de São Paulo

**Keywords:** temporal bone, facial paralysis, peripheral primitive neuroectodermal tumor

## INTRODUCTION

The primitive neuroectodermal tumor (NEDT) was first described in 1973 by Hart and Earle in order to characterize an undifferentiated neoplasia of the central nervous system, which represents between 90 and 95% of undifferentiated cells and does not fulfill diagnostic criteria for any other type of neoplasia.^1^,^2^ The peripheral NEDT on the head and neck is extremely rare, and in a recent literature review we did not see any publication of such tumor involving the temporal bone and the facial nerve canal.^3^

Our goal with the present paper is to report on a patient who presented peripheral facial paralysis as initial manifestation of the peripheral NEDT involving the temporal bone.

## CASE REPORT

**Figure 1:**
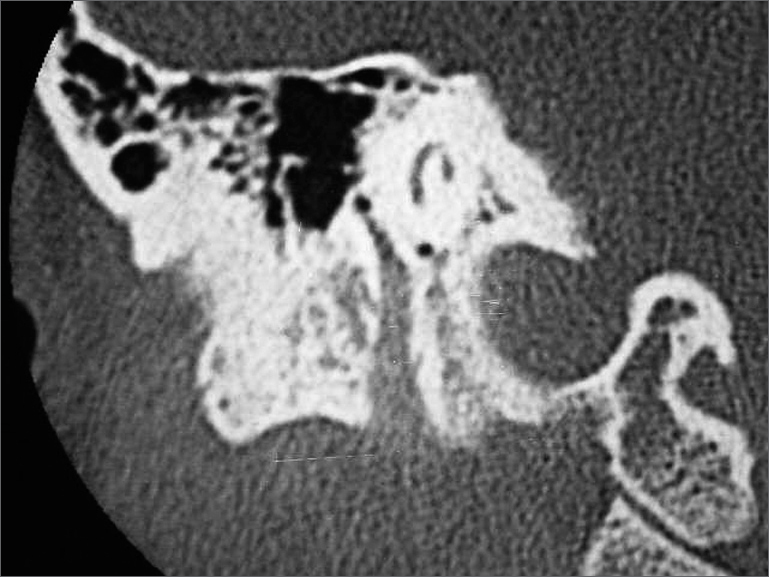
Coronal temporal bone CT scan, showing an image with soft tissue density, occupying and enlarging the third portion of the facial nerve, all the way to stylo-mastoid foramen.

R.A.O., a 32 year old female, complaining of 30 days of facial movement impairment, associated to right side otalgia. Otolaryngological exam showed grade IV (House-Brackmann scale) peripheral facial paralysis on the right side, and normal otoscopic exam. Temporal bone CT scan showed enlargement of the facial nerve canal within the mastoid (Figure 1). Following that, we ordered a brain and skull base MRI study; the patient returned after three weeks with a normal brain MRI and additional complaints (back pain and lower limb paresis), being admitted to the Neurosurgery department. Through image studies (CT scan and MRI of the spine) we saw an osteolytic lesion on the tenth thorax vertebrae (T10), with vertebral canal invasion. Chest x-ray showed a nummular image on the right hemithorax. A fine needle biopsy was carried out on the T10 lesion, which determined, through immunohistochemistry analysis (positive Vimentin and Mic2/CD99), the diagnosis of NEDT. Despite chemotherapy treatment with vincristine, cyclophosphamide and adriablastin, the patient passed away on the sixth month of follow up.

## DISCUSSION

Temporal bone and facial nerve involvement by neoplasia is not common, it represents about 5% of peripheral facial paralysis causes^4^, and malignant tumors are even rarer. Temporal bone metastases are extremely rare, not even having a notified real incidence.

Peripheral NEDT is a neoplasia of primitive neuroectodermal cells, of rare occurrence. It comes from the peripheral nervous system and bears considerable predilection for limbs and pelvis, regardless of age range.^5^ Most NEDT cases that involve the spinal cord represent metastatic lesions of an intracranial primary tumor; however, the spine may be its primary site, especially in young adults^2^. In this case, it is very likely that the primary lesion started on the facial nerve, followed by metastasis to the spinal cord and lung, as we can see in the clinical evolution of symptoms. We believe the lesions located in the lung and the spinal cord to be metastasis, because in blood borne spread these are the preferred sites due to their intense vascularization.

In the literature we found numerous series showing varied primary sites affected by the NEDT.^3^,^6^ It is thought that the NEDT comes from the neoplastic transformation of primitive neuroepithelial cells. The fact that such cells may remain anywhere in the nervous system may explain the diverse origins of this tumor.^2^

In our bibliographic review (MEDLINE and LILACS) we found no report of temporal bone involvement by NEDT primary or metastatic lesions. Diagnostic confirmation for this tumor is carried out through immunohistochemistry, and it is positive for Vimentine^3^ and Mic2/CD99.^2^,^3^ Despite combined treatment (surgery, chemo and radiotherapy) prognosis is still poor, with average survival between 6 and 42 months.^2^

## FINAL REMARKS

Peripheral NEDT is an extremely rare neoplasia, with very few cases described in the literature and without a definitive clinical presentation pattern. This is the first literature report of peripheral facial paralysis as initial NEDT manifestation by involvement of the temporal bone.
